# Household Hints and Recipes

**Published:** 1884-01

**Authors:** 


### Household Hints & Recipes.

A bronze which resists oxidation and is especially fitted for mirrors and reflectors, consists of fifty-two parts of copper, thirty parts of nickel, twelve parts of zinc, five parts of lead and one part of bismuth.

A DISINFECTANT LAUNDRY BLUE—Mix together sixteen parts of Prussian blue, two parts of carbolic acid, one part of borax and one part of gum arabic into a stiff dough. Roll it out into balls as large as hazel nuts, and coat them with gelantine or gum, to prevent the carbolic acid from escaping.

LABEL VARNISH—One of the best label varnishes is the following: Sandarac (in coarse powder). 100 parts; mastic, forty parts; copaiba fifteen parts; venice turpentine, thirty parts; oil of turpentine, forty parts; alcohol, ninety parts; absolute alcohol, ninety parts. Macerate until solution is effected.

RESTORING THE FINISH OF LEATHER—Leather may be restored in color, if not too far gone, by a slight application of oil. If this is not effectual, put on blacking, let it dry, brush it off, and go over it again very lightly with oil. If very brown, black thoroughly and oil it afterwards, giving it a final dressing of dissolved gum tragacanth.—*Furniture Gazette.*

TO KEEP WOODEN POSTS FROM ROT—A practical farmer, and one who has thoroughly tested the matter, says that wooden posts will last in the ground even longer than iron if treated as follows: Take boiled linseed oil and stir in pulverized coal to the consistency of paint. Put a coat of this over the timber, and there is not a man will live to see it rot.

THOMAS'S ELECTRIC OIL—L. L. Briggs, of New Hampton, Iowa, communicates the following receipt to Stearns' *New Idea*: Gum camphor, one-half ounce; oil of gaultheria, one-half ounce; oil of origanum, one-half ounce; chloroform, one ounce; laudanum, one ounce; oil of sasafras, one ounce; oil of hemlock, one ounce; oil of turpentine, one ounce; balsam fir, one ounce; tincture of guaicum, one ounce; tincture of catechu, one ounce; alcohol, four pints. Alkanet sufficient to color.

IMITATION WALNUT—We have it on good authority that an excellent stain for giving light-colored wood the appearance of black walnut may be made and applied as follows: Take Brunswick black, thin it down with turpentine until about the right tone and color, and then add about one-twentieth its bulk of varnish. This mixture, it is said, will dry hard and take varnish well.

PRESERVATION OF FRUIT—To preserve fruit fresh for a long time—it is said for years—it may be placed into sand which has previously been carefully washed, dried and then dampened with cognac. Care must be taken that the fruits, which should not be over-ripe nor unripe, do not approach each other too closely. The earthen or wooden vessel in which the sand is contained must be kept well covered in a place which is neither too damp nor too warm.—*Rundschau Leitmeritz.*

GRAPE LEAVES FOR PICKLES—A writer in the *Country Gentleman* recommends the use of fresh green grape leaves to place on top of pickles in jars in place of flannel or other cloth usually employed. He claims the leaves will preserve the vinegar sharp and clear and impart a nice flavor. The leaves should be rinsed in pure water and left to drain before use, and occasionally changed. They exclude the air, and besides imparting a delightful flavor to the pickles cause less trouble to the housewife.

BLACK LACQUER FOR COATING BOTTLES—Bottles, or other glass vessels, which it is desired to make impervious to light, may be coated, according to Ferd Simand, with a black lacquer prepared in the following manner: Equal part of asphalt and of boiled linseed oil are heated for one hour, over a naked fire to about 200° C. (392° F.); then a sufficient quantity of lamp-black, previously triturated with oil of turpentine, is added to make a mixture, which, when mixed with one-fourth or one-third its volume of oil of turpentine, will cover well. Usually, one coat is sufficient; in special cases, two coats may be required.

Sometimes it is desireable to be able to see the height at which the liquid in the bottle is standing. This may be accomplished, according to the author, by leaving a small round spot on opposite sides uncoated. The bottom of the bottle is likewise left unvarnished.—*Dingl. Pol. Journ.*

TO CLEAN BRASS—The government method prescribed for cleaning brass, and in use at all the United States arsenals, is claimed to be the best in the world. The plan is to make a mixture of one part common nitric acid and one-half part sulphuric acid, in a stone jar, having also ready a pail of fresh water and a box of sawdust. The articles to be treated are dipped into the acid, then removed into the water, and finally rubbed with sawdust. This immediately changes them to a brilliant color. If the brass has become greasy, it is first dipped in a strong solution of potash and soda in warm water; this cuts the grease, so that the acid has free power to act.

SHAVING SOAP—The following method of preparing good shaving soap by the cold process is given by Kurten in the *Seifensieder Zeitung.* Melt together forty parts of fresh white tallow and twenty parts of cocoanut oil at a temperature of 113° Farh. Gradually stir into this thirty four parts of a thirty per cent. solution of caustic soda and six parts of a thirty per cent. potash solution. The mixture is now stirred fifteen or twenty minutes until it forms a uniform soapy mass. When a scum forms on the surface it is a sign that the saponification is complete. It may be perfumed with one-fifth part of oil of caraway, one-fourth part of bergamot, one-eighth each of thyme and lavender. This soap contains no free alkali, and gives a good lather.

NICKEL PLATING—Nickel can be separated by the galvanic current from any neutral solution; but, as soon as the liquid becomes acid (owing to the withdrawal of the base), the reaction ceases. To obviate this, an arrangement is made to cause the liquid to preserve its neutrality constantly. This is accomplished by using an anode (or pole) made of metalic nickel. It has also been found that the best nickel-salt for plating purposes is the double sulphate of nickle and ammonium, which is easily obtained pure. This salt is dissolved to saturation in pure water, at a temperature between 20° and 25° C., (68°–77° F.); and this temperature, which is the most suitable for preducing a uniform and firm deposit, must be maintained during the whole process. Another good plating liquid is that proposed by Powell, namely, 90 gm. of sulphate nickel, 25 gm. of citrate of nickel, 15 gm. of benzoic acid and 4 litters of water. The nickel anodes should be selected of as large a surface as possible, particularly if the galvanic current is weak.

Brass may be hickel-plated, according to Stolba, without a battery, by boiling first in a solution of chloride of zinc containing metallic zinc, and then adding a soluable nickel-salt.

TO EMBALM SMALL QUADRUPEDS—R. J. Gorriz Y. Munoz recommends, in the *Restaurador Farmaceutico*, the following convenient method: First an opening is made from the os pubis to the anus, through which the abdominal and pectoral cavities are emptied. The interior is next painted by means of a camel-hair brush, with a ten per cent. alcoholic solution of carbolic acid. After a couple of hours it is dusted thoroughly with powdered burnt alum, and stuffed with cotton wadding sprinkled with the same carbolic solution, to which one per cent. of corrosive sublimate has been added. The cavity being well filled, the opening is sewn up, and a little of the carbolic solution is introduced into the ears, mouth and nostrils. It is also well, although not absolutely necessary, to moisten with the same the whole skin, unless a solution of alum be applied as a tanning material. The specimen now only requires drying in the shade, and in a few days it may be placed in a convenient place in the cabinet.

PRIZE RECEIPT FOR SALAD—From six or eight lettuces, Cos or cabbage, removing outer or coarse leaves, and strip from remaining ones the good part. The pieces should be two and a half to three inches long, and may be broken up, but not cut; then wash them and let them remain about half an hour in water. Rinse in second water, place in napkin, and swing till dry. For dressing take the yolks of two hard-boiled eggs, crush them to paste in bowl, adding half tablespoonful French vinegar, three mustard-spoons mustard, one salt-spoon salt, and beat up well together; then add, by degrees, six to eight tablespoonfuls Lucca or Provence oil, one of vinegar, and, when thoroughly mixed, a little tarragon finely chopped, a dessert-spoon coarse white pepper, as pepper in powder irritates the palate. When it is mixed, place the salad in it, and turn over and over, thoroughly and patiently, till there remains not one drop of liquid at bottom of bowl. Put the white of the eggs in slices on the top and serve shortly after it is mixed.—*Labouchere, in London Truth.*

